# Quantification of dual-energy CT-derived functional parameters as potential imaging markers for progression of idiopathic pulmonary fibrosis

**DOI:** 10.1007/s00330-021-07798-w

**Published:** 2021-03-16

**Authors:** Sarah C. Scharm, Jens Vogel-Claussen, Cornelia Schaefer-Prokop, Sabine Dettmer, Lars Knudsen, Danny Jonigk, Jan Fuge, Rosa-Marie Apel, Tobias Welte, Frank Wacker, Antje Prasse, Hoen-oh Shin

**Affiliations:** 1 Institute of Diagnostic and Interventional Radiology, Hannover Medical School, Carl-Neuberg-Str.1, 30625 Hannover, Germany; 2 Biomedical Research in Endstage and Obstructive Lung Disease Hannover (BREATH), German Center for Lung Research, Hannover, Germany; 3 Department of Radiology, Radboud University, Nijmegen, The Netherlands; 4 Department of Radiology, Meander Medical Center, Amersfoort, The Netherlands; 5 Institute of Functional and Applied Anatomy, Hannover Medical School, Hannover, Germany; 6 Institute of Pathology, Hannover Medical School, Hannover, Germany; 7 Department of Respiratory Medicine, Hannover Medical School, Hannover, Germany

**Keywords:** Idiopathic pulmonary fibrosis, Multidetector computed tomography, Image processing, computer-assisted, Pulmonary ventilation, Perfusion imaging

## Abstract

**Objectives:**

The individual course of disease in idiopathic pulmonary fibrosis (IPF) is highly variable. Assessment of disease activity and prospective estimation of disease progression might have the potential to improve therapy management and indicate the onset of treatment at an earlier stage. The aim of this study was to evaluate whether regional ventilation, lung perfusion, and late enhancement can serve as early imaging markers for disease progression in patients with IPF.

**Methods:**

In this retrospective study, contrast-enhanced dual-energy CT scans of 32 patients in inspiration and delayed expiration were performed at two time points with a mean interval of 15.4 months. The pulmonary blood volume (PBV) images obtained in the arterial and delayed perfusion phase served as a surrogate for arterial lung perfusion and parenchymal late enhancement. The virtual non-contrast (VNC) images in inspiration and expiration were non-linearly registered to provide regional ventilation images. Image-derived parameters were correlated with longitudinal changes of lung function (FVC%, DLCO%), mean lung density in CT, and CT-derived lung volume.

**Results:**

Regional ventilation and late enhancement at baseline preceded future change in lung volume (*R* - 0.474, *p* 0.006/*R* - 0.422, *p* 0.016, respectively) and mean lung density (*R* - 0.469, *p* 0.007/R - 0.402, *p* 0.022, respectively). Regional ventilation also correlated with a future change in FVC% (*R* - 0.398, *p* 0.024).

**Conclusion:**

CT-derived functional parameters of regional ventilation and parenchymal late enhancement are potential early imaging markers for idiopathic pulmonary fibrosis progression.

**Key Points:**

*• Functional CT parameters at baseline (regional ventilation and late enhancement) correlate with future structural changes of the lung as measured with loss of lung volume and increase in lung density in serial CT scans of patients with idiopathic pulmonary fibrosis.*

*• Functional CT parameter measurements in high-attenuation areas (- 600 to - 250 HU) are significantly different from normal-attenuation areas (- 950 to - 600 HU) of the lung.*

*• Mean regional ventilation in functional CT correlates with a future change in forced vital capacity (FVC) in pulmonary function tests.*

## Introduction

The aim of antifibrotic drug treatment is to slow down or at best stabilize the progression of the disease. The individual course of disease in idiopathic pulmonary fibrosis (IPF) is highly variable [1]. Prospective assessment of disease activity and early categorization of patients with fast as opposed to those with no or slow progression could optimize individual therapy management.

Imaging with high-resolution computed tomography plays a central role in diagnosing and monitoring interstitial lung disease [2–4]. However, the visual evaluation of the extent and progression of disease on CT relies on the assessment of morphology rather than function, is cumbersome, and is prone to interobserver variability [2, 5]. A number of computerized tools for more objective and reproducible measurements have been developed over recent years. They include analysis of density histograms, including mean density, skewness, and kurtosis [6–8]. Texture-based methods consider different patterns and provide superior information if analyzed on a regional rather than global base [9, 10].

Several studies showed that quantification of density-based histogram parameters and quantification of various patterns of fibrosis correlate well with lung function parameters at the time of imaging, and some of them were predictive of mortality [7, 11]. These methods, however, are based on morphological findings only and the fact that measurable structural changes over time already occurred.

Up to now, there is limited experience in using CT for the evaluation of pulmonary ventilation and perfusion in the context of diffuse lung disease.

We designed a dual-energy CT acquisition protocol that provides information about ventilation, perfusion, and late enhancement (VPL-CT) in addition to the usual imaging of morphology [12]. The adopted CT protocol in inspiration/expiration was modified in two ways: it required the injection of contrast, and the expiration scan was acquired with a 5-min delay. Subsequently, CT data underwent advanced processing to allow for regional (voxel-based) ventilation and perfusion analysis.

The aim of this study was to investigate whether CT-derived functional parameters can serve as early functional imaging markers for disease progression in patients with IPF prior to the change of established morphologic CT parameters (decrease of lung volume[13] and increase of mean lung density [6, 14]) and decline of lung function parameters (FVC% and DLCO%).

## Methods

### Study group

The retrospective study was approved by the internal review board (No. 3649-2017), and written consent was received from all patients. Between September 2016 and April 2020, all patients with (histologically or major diagnostic categories (MDC-)) confirmed diagnosis of IPF were included, who underwent two dual-energy CT scans with a time interval of at least 6 months. At the time of CT acquisition, none of the patients suffered from pulmonary comorbidities (e.g., pneumonia, acute exacerbation, decompensation of cardiac function with pulmonary edema) as ruled out clinically and by visual analysis of the CT scans by an experienced chest radiologist (H.-O.S.).

### CT data acquisition

CT examinations were performed with a dual-source CT (Somatom Force®, Siemens Healthineers) at 90 kV/150 kV in full inspiration and maximum expiration (mean CTDI 5.52 ± 1.88 mGy for inspiration / mean CTDI 4.13 ± 1.43 mGy for expiration). Scans were obtained with patients in a supine position and without respiratory gating. Patients were instructed and trained to cooperate with the scan protocol as best as possible.

After intravenous administration of a 60-mL contrast agent (Iomeron 400, Bracco), the inspiration scan was triggered when the intraluminal density within the left ventricle reached 200 HU with a delay of 12 s. The expiration scan was performed with a 5-min delay. Virtual non-contrast (VNC) and pulmonary blood volume (PBV) images were generated for both breathing positions using dedicated software (syngo.via® v 5.1, Siemens Healthineers). For CT reconstruction, an iterative algorithm (ADMIRE), a soft tissue kernel (Qr40), and a reconstruction interval of 1 mm were applied.

### Image processing

All functional parameters were calculated per CT-voxel with transversal dimensions of 512 × 512 voxels. Applying a field of view of 35 cm, the voxel size was 0.68 × 0.68 × 1 mm. The following processing steps were performed:

1. VNC and PBV images were generated for both respiratory positions by material decomposition [15].
2. Automatic lung segmentation was performed on the VNC images using in-house software based on a deep learning algorithm [16].
3. To morphologically align the inspiration and expiration images, a non-linear registration was performed, warping the inspiration scan to the expiration scan. The registration was performed using an open-source software package (ANTS, 2011, Release 1.5, Penn Image Computing and Science Laboratory).

#### Calculation of lung perfusion and late enhancement

4. The inspiration PBV was regarded as a surrogate for pulmonary parenchymal perfusion assessing the amount of iodine per voxel as measured by attenuation increase relative to the VNC image [17].

The delayed expiration scan was used for “late enhancement” imaging as a surrogate marker for slow washout of contrast media of the lung parenchyma due to alveolar-capillary leakage and increased interstitial volume. The values for lung perfusion and late enhancement were normalized to the mean of the contrast in the pulmonary artery trunk and the ascending aorta.

Thus, the lung perfusion and late enhancement values were relative, reflecting their percentage of the mean contrast in the named vessels at the respective scan time.

#### Calculation of lung ventilation

5. For calculation of the regional ventilation, the Inverse Jacobian determinant of the deformation field was used to calculate the voxel-wise volume change between full inspiration and full expiration. The difference in voxel density in inspiration and expiration was used as a correction factor for calculating the specific air volume change [18]. Normalization to the inspiration scan yielded the following equation:
  $$ \mathrm{Regional}\ \mathrm{ventilation}=1-\Big(\mathrm{Inverse}\ \mathrm{Jacobian}\times \left(\frac{\mathrm{lung}\ \mathrm{density}\left(\mathrm{expiration}\right)}{\mathrm{lung}\ \mathrm{density}\left(\mathrm{inspiration}\ \mathrm{warped}\right)}\right) $$

The Inverse Jacobian was used since it depicted the shrinkage from the inspiration scan to the expiration scan. The regional ventilation values range from 0 (no volume change at all) to 1 (total collapse in expiration) and represent the voxel-wise air volume change between inspiration and expiration. Values between 0.4 and 0.5 have been described for healthy subjects [19, 20].

#### Definition of functional lung tissue

The functional lung tissue on CT was defined in the inspiration scan by applying a mask with values between - 950 and - 250 HU. The lower threshold was chosen to exclude low attenuation areas (LAA), e.g., emphysema and air in honeycombing [21]. The upper threshold was set to include both normal lung tissue with attenuation values between - 950 and - 600 HU (NAA) and high-attenuation areas (HAA) with attenuation values between - 600 and - 250 [21, 22]. The latter represented lung parenchyma with signs of fibrosis (ground glass, thickened intra- and interlobular septae, leading to an increased lung density). The calculation of functional CT parameters was restricted to the area of functional lung tissue. Intrapulmonary vessels were excluded.

**Fig. 1:**
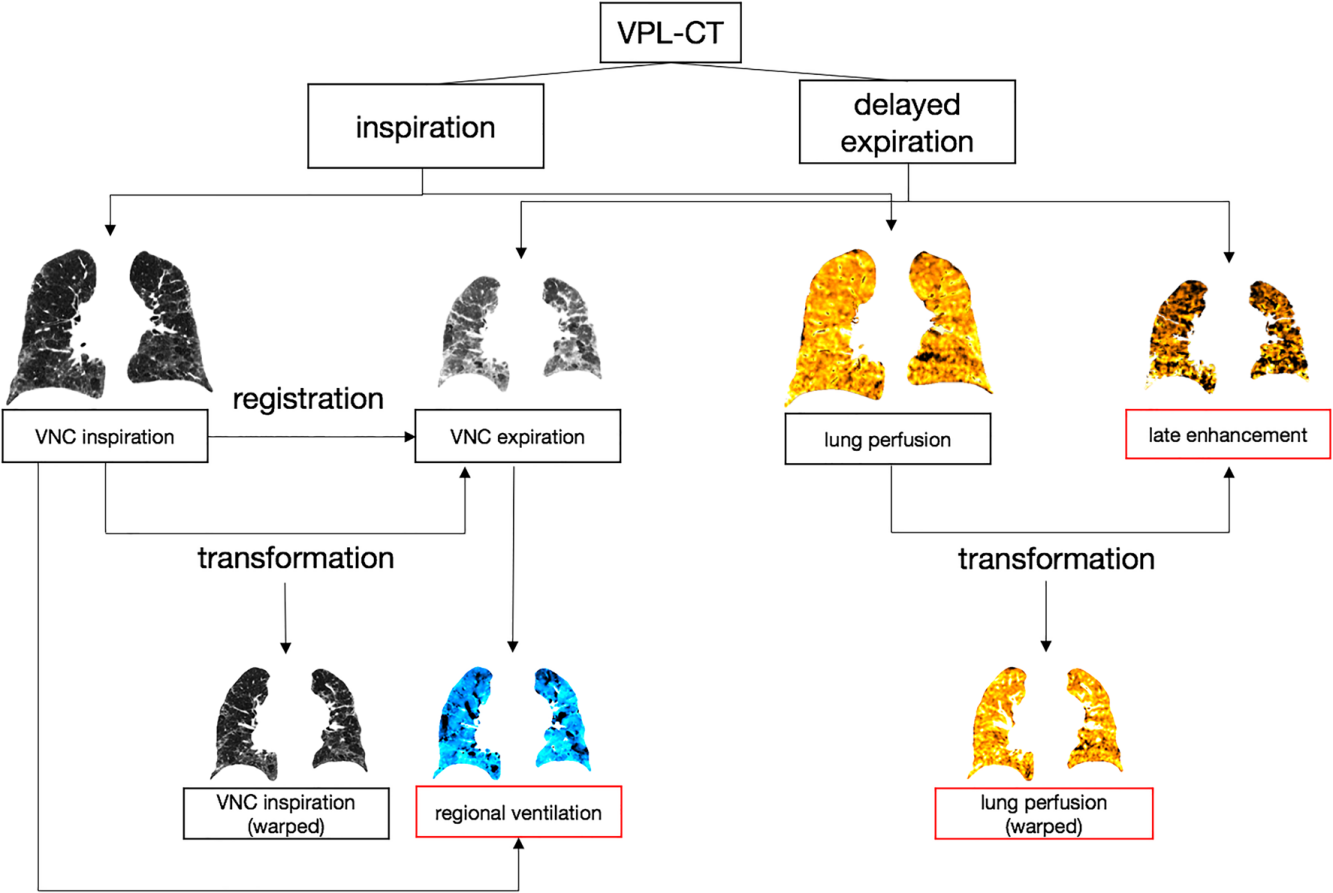
Ventilation-perfusion-late enhancement CT (VPL-CT) Ventilation, lung perfusion, and late enhancement images were calculated from the VPL-CT. The VNC images in inspiration were registered to the VNC images in expiration. The VNC inspiration and lung perfusion images were transformed to match the expiration scan (VNC inspiration warped and lung perfusion warped, respectively). The inverse Jacobian determinant was used to calculate the regional ventilation from the VNC images in inspiration and expiration. The intensities of the CT-derived functional parameter images are illustrated in color

Image processing resulted in five aligned datasets per VPL-CT scan (Fig. 1).

### Pulmonary function tests

All patients underwent a body plethysmography measurement according to the guidelines of the European Respiratory Society at a maximum time lag of 2 months to the corresponding CT scan.

FVC% and DLCO% at baseline and the ratio follow-up/baseline were used for the statistical analysis.

### Statistical analysis

#### Quantification of functional CT parameters at baseline

Mean regional ventilation, lung perfusion, and late enhancement at baseline were quantified, calculating the mean values for the functional lung tissue as well as for NAA and HAA areas, separately. The significance of differences between the functional CT parameters in the respective areas was tested using a paired *T* test.

#### Correlation analysis

Disease progression for the correlation analysis was determined by the change of lung volume, the mean lung density, and the change of the PFT parameters FVC% and DLCO% over time. The lung volume and the mean lung density were calculated from the CT images using a segmentation mask.

The mean values of regional ventilation, perfusion, and late enhancement at baseline CT were correlated with the PFT parameters, CT lung volume, and mean lung density at baseline, as well as with the change of these values over time using the follow-up/baseline ratios.

The PFT parameters, the lung volume, and the mean lung density of the follow-up examination were normalized to a period of 1 year for statistical analysis.

The Pearson correlation with a significance level of *p* ≤ 0.05 was applied.

The statistical programs used were SPSS Statistics 25 (IBM) and MATLAB R2019a (MathWorks). For visualization purposes, the open-source package Fiji (https://imagej.net/Fiji) was used.

## Results

### Study group

**Fig. 2:**
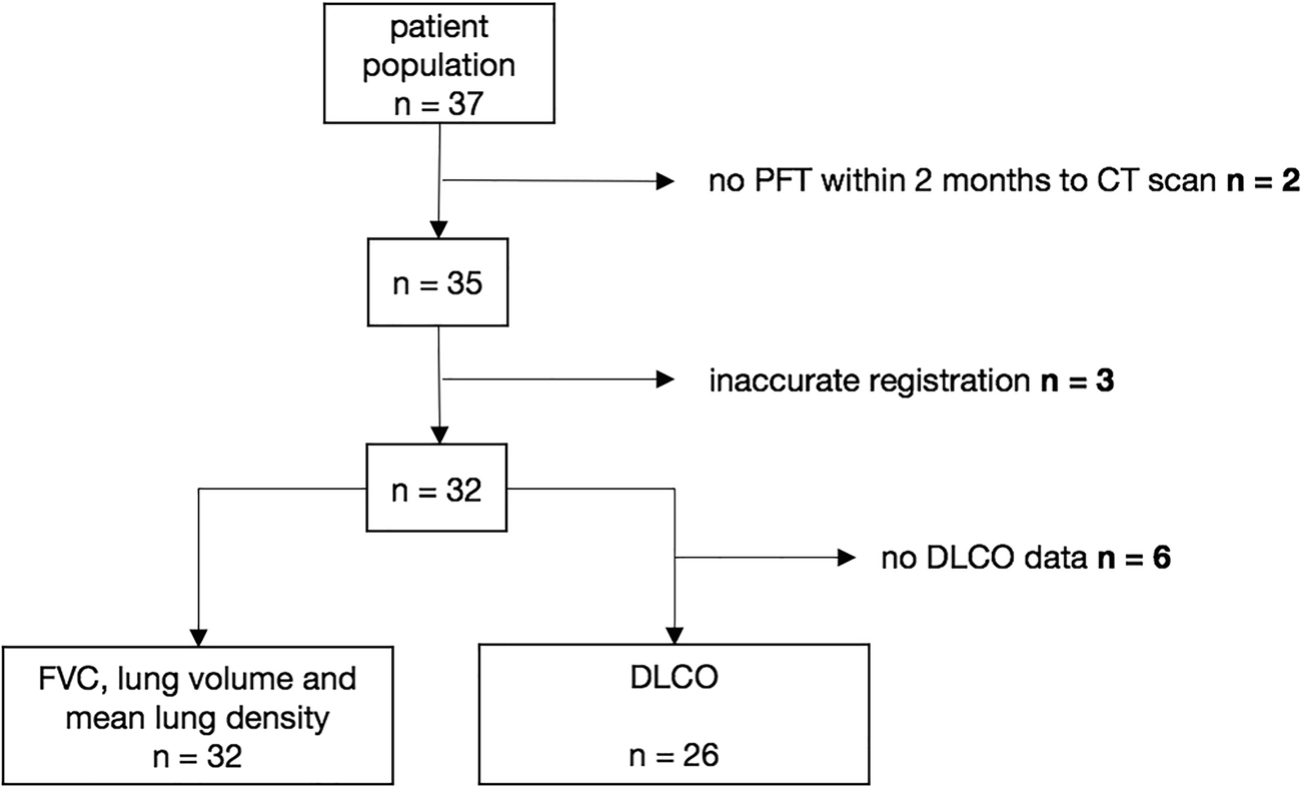
Flowchart depicting exclusion criteria. In 2 of the 37 patients primarily included, no PFT was performed within 2 months after the CT examination. Three patients were excluded due to registration errors. The FVC, lung volume, and mean lung density measurements were available for the remaining 32 patients. For 6 patients, the DLCO values were missing

The final study group consisted of 32 patients. Five of the 37 included patients had to be excluded from the final analysis since their PFT exceeded the maximum time interval of 2 months to the corresponding CT scan (*n* = 2) or due to technical errors (*n* = 3, inaccurate registration of CT data with misalignment exceeding 2 mm). The DLCO analysis was limited to 26 patients (Fig. 2).

Patient population characteristics are summarized in Table 1.

**Table 1:** Patient population characteristics. The population predominantly consisted of elderly men. Most patients were under antifibrotic treatment

Parameter	No.
No. of patients	32
Sex	
Men	28
Women	4
Age (years)	70 ± 10
Smoking status	
Never	13
Former	18
Current	1
Antifibrotic medication	21
Mean time between baseline and follow-up CT (months)	15.4 ± 6.7
Mean time between CT and PFT at baseline (days)	14 ± 13
Mean time between CT and PFT at follow-up (days)	13 ± 15

### Reference parameters

The mean FVC%, DLCO%, lung volume, and mean lung density values of baseline and follow-up are depicted in Table 2.

**Table 2:** Reference parameters at baseline and follow-up. All parameters were significantly different (*p* ≤ 0.05) between the two time points

Reference parameters	Baseline	Follow-up	
	Mean ± SD	Mean ± SD	*p*
FVC%	69.75 ± 17.25	64.43 ± 15.98	0.009
DLCO%	52.58 ± 14.55	43.76 ± 12.36	0.000
lung volume (in mL)	3942.09 ± 844.33	3719.76 ± 869.13	0.001
mean lung density (in HU)	- 742.23 ± 51.05	- 728.30 ± 62.30	0.016

### CT-derived functional parameters

At baseline, the mean values of regional ventilation, lung perfusion, and late enhancement in the functional lung tissue, averaged over all patients, were 0.63 ± 0.10, 9.02 ± 2.12, and 38.00 ± 11.60, respectively. The functional lung volume represented, on average, 96% of the total lung volume in inspiration.

**Fig. 3:**
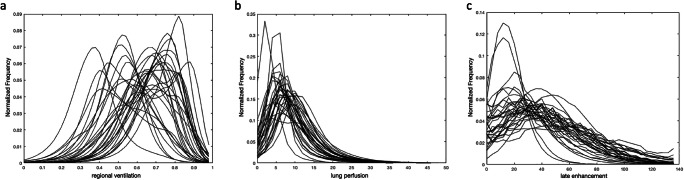
Histograms of functional CT parameters. **a** Regional ventilation (fractional air volume change between inspiration and expiration), (**b**) lung perfusion (percentage of vessel contrast), and (**c**) late enhancement (percentage of vessel contrast in the delayed scan)

Figure 3 shows the frequency distributions of regional ventilation, lung perfusion, and late enhancement of functional lung tissue for each patient. Regional ventilation (Fig. 3a) showed the most considerable variation among the patients, with most patients showing regions with unphysiologically high values above 0.75, some even showing mean values around 0.75. Late enhancement varied to a lesser extent (Fig. 3c), while perfusion showed the smallest difference between patients (Fig. 3b). In some lung regions, late enhancement values above the vascular contrast (> 100%) were seen.

For the comparison of the CT-derived functional parameters in NAA and HAA, the following averaged mean values ± SD were found in the respective density areas: regional ventilation NAA/HAA: 0.62 ± 0.10/0.66 ± 0.10; lung perfusion NAA/HAA: 8.35 ± 2.04/12.28 ± 3.00; late enhancement NAA/HAA: 33.96 ± 10.62/55.42 ± 14.66.

**Fig. 4:**
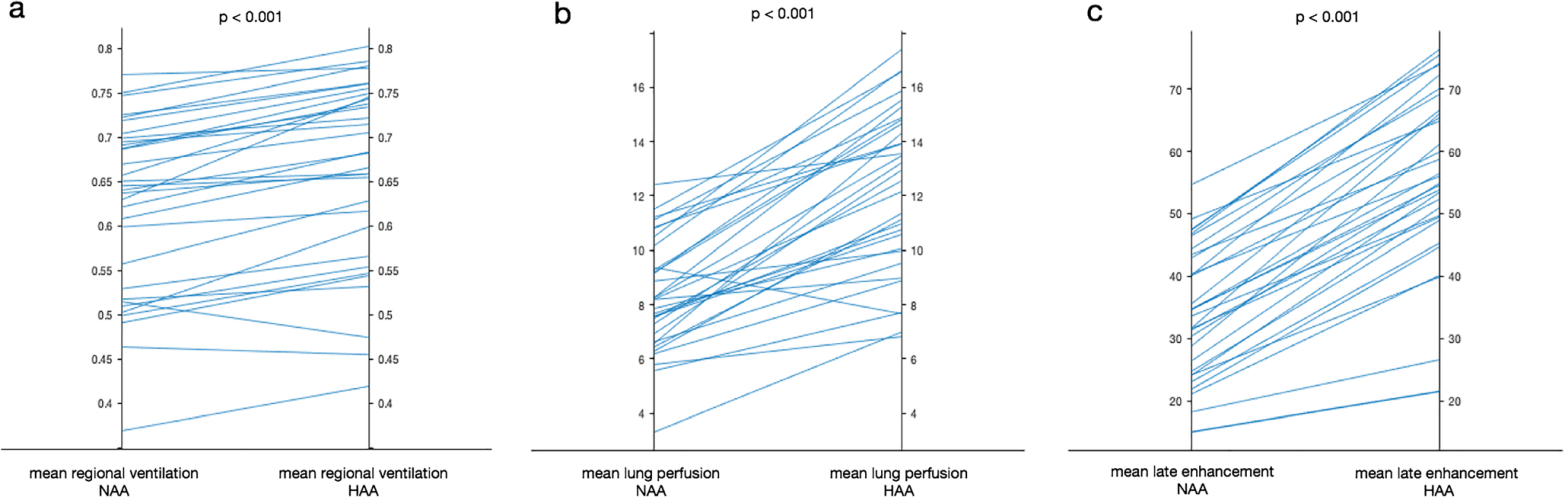
Functional CT parameters in different density areas. Mean values of regional ventilation (**a**), lung perfusion (**b**), and late enhancement (**c**) compared in normal-attenuation areas (- 950 to - 600 HU) and high-attenuation areas (- 600 to - 250 HU) for all patients

Figure 4 illustrates the differences between NAA and HAA for each patient.

The mean values of regional ventilation (*p* < 0.001), lung perfusion (*p* < 0.001), and late enhancement (*p* < 0.001) significantly differed in NAA and HAA. All three functional parameters were higher in HAA than in NAA.

In inspiration, NAA was, on average, 80% and HAA 15% of the total lung volume.

#### Correlation of functional CT parameters with signs of progressive fibrosis

In the absence of other acute pulmonary comorbidities, a decrease in mean lung volume [13] and an increase in mean lung density [6, 14]—both derived from CT—as well as a decline in PFT were considered indicators of progressive fibrosis.

### Ventilation

Mean regional ventilation at baseline showed negative correlations with the ratios (follow-up/baseline) of lung volume (*R* - 0.474, *p* 0.006), of the mean lung density (*R* - 0.469, *p* 0.007), and of the FVC% (*R* - 0.398, *p* 0.024) thus showing a correlation with progression of fibrosis.

### Lung perfusion

The mean lung perfusion at baseline did not correlate with any ratios of the reference values. However, a negative correlation was found between the baseline lung volume (*R* - 0.582, *p* 0.000) and the baseline FVC% (*R* - 0.490, *p* 0.004), and a positive correlation with the baseline mean lung density (*R* 0.523, *p* 0.002) indicating a correlation with the extent of fibrosis at baseline.

### Late enhancement

The mean late enhancement at baseline showed a negative correlation with lung volume and a positive correlation with mean lung density, both at baseline (*R* - 0.490, *p* 0.004/*R* 0.570, *p* 0.001). Secondly, it correlated negatively with the ratios of lung volume and mean lung density (*R* - 0.422, *p* 0.016/*R* - 0.402, *p* 0.022). These results indicate a correlation of the late enhancement with both the baseline extent and future progression of fibrosis.

**Fig. 5:**
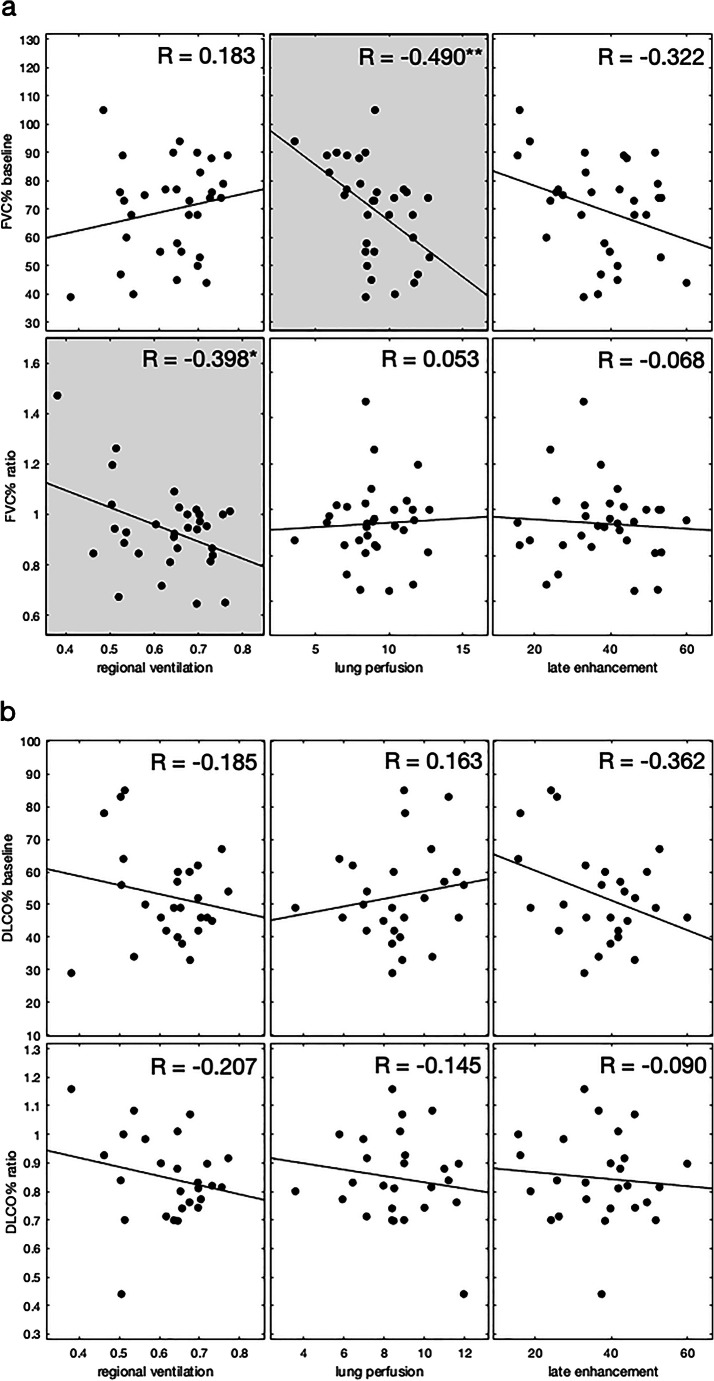

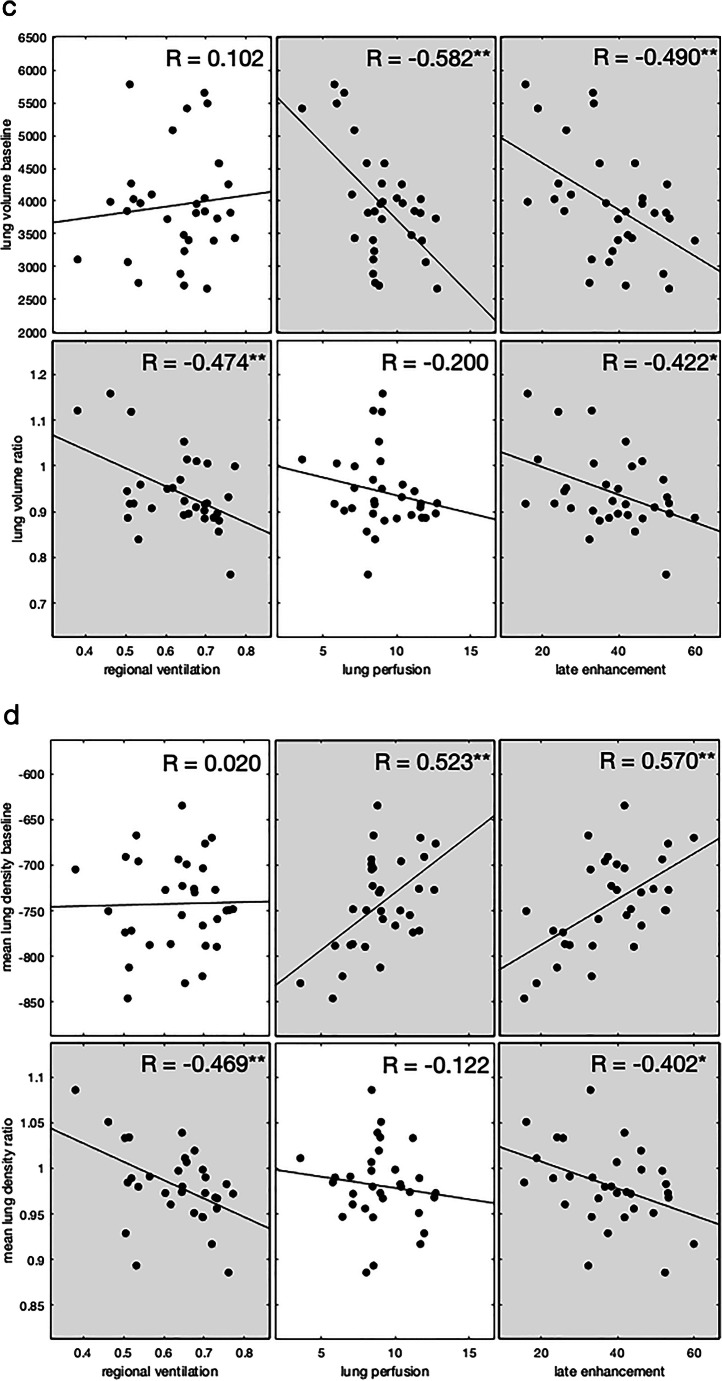
Scatterplots of the correlation analysis. Regional ventilation, lung perfusion, and late enhancement vs baseline values and ratios (follow-up/baseline) of FVC% (**a**), DLCO% (**b**), lung volume (**c**), and mean lung density (**d**). Significant correlations are highlighted in grey (*p < 0.05, **p < 0.01). Least-square lines are superimposed on each plot

**Fig. 6:**
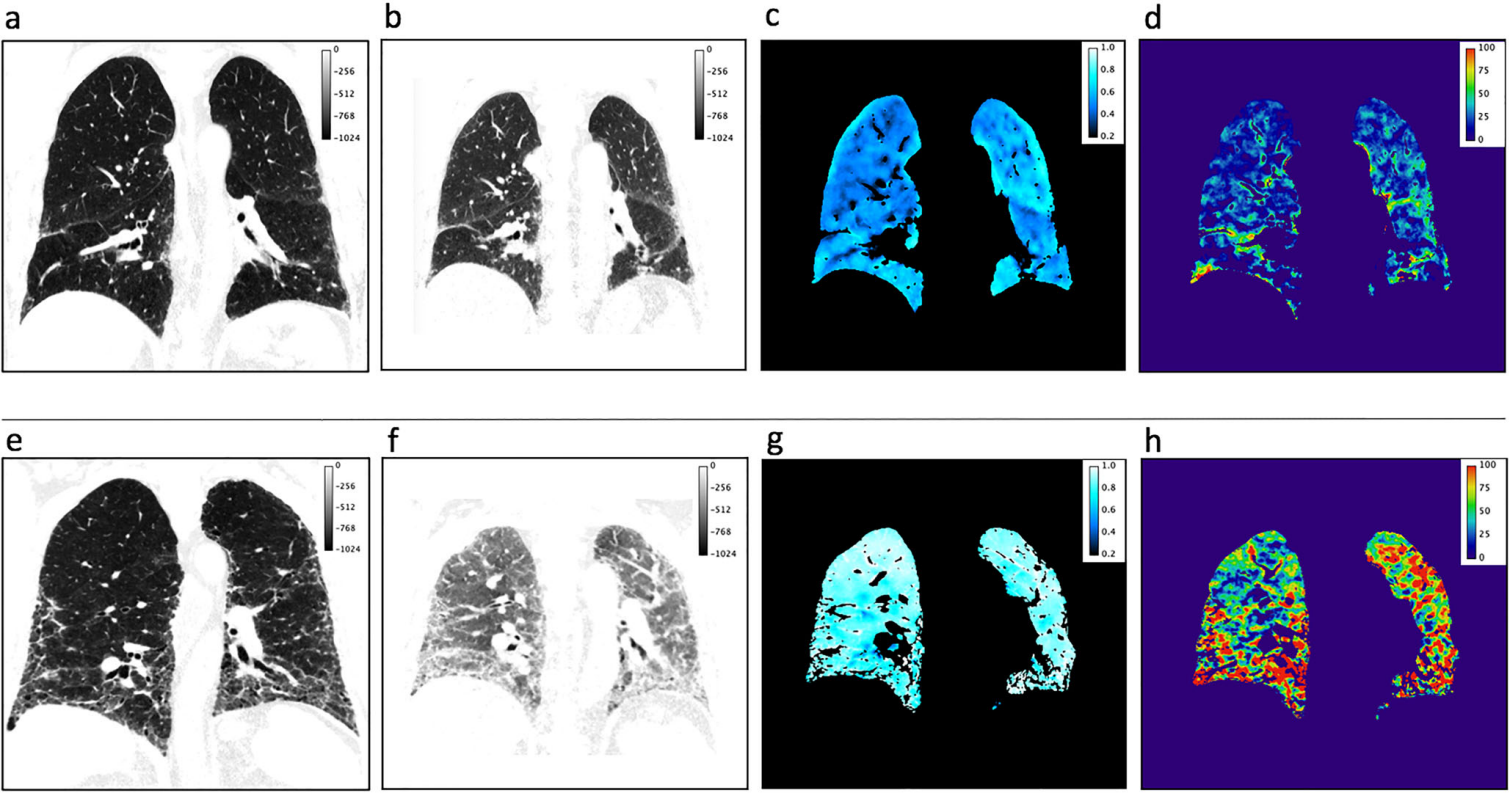
Baseline scans of a patient with stable disease (upper row, **a**–**d**) and a patient with progressive disease (lower row, **e**–**h**). The images are presented in the following order: CT in inspiration, CT in expiration, regional ventilation, and late enhancement. *Stable patient* mean regional ventilation 0.51; mean late enhancement 24%; baseline/follow-up values FVC% 73%/85%; lung volume 4269 mL/ 4549 mL; mean lung density - 812 HU/-827 HU. *Progressing patient* mean regional ventilation 0.76; mean late enhancement 52%; baseline/follow-up values FVC% 79%/57%; lung volume 3823 mL/ 3058 mL; mean lung density - 749 HU/− 677 HU. The patient in the upper row showed a low disease activity, as indicated in the regional ventilation and late enhancement images. FVC% and the mean lung volume and density values even improved with antifibrotic therapy. The patient in the lower row presented much higher ventilation and late enhancement values in the baseline scan, resulting in disease progression in the follow-up’s reference parameters

Table 3 shows the results of the correlation analysis and Fig. 5 the corresponding scatterplots. Figure 6 depicts the baseline CT scans of two patients with varying disease progression.

**Table 3:** Correlation analysis results. Longitudinal correlation of functional CT parameters with FVC%, DLCO%, segmented lung volume, and mean lung density. Significant results are italicized; * *p* < 0.05, ** *p* < 0.01; *b*, baseline; *f*, follow-up; *f/b*, ratio follow-up/baseline

Reference parameters	Mean regional ventilation		Mean lung perfusion		Mean late enhancement	
	*R* value	*p* value	*R* value	*p* value	*R* value	*p* value
FVC % b	0.183	0.316	−0.490**	0.004**	−0.322	0.072
FVC % f/b	−0.398*	0.024*	0.053	0.773	−0.068	0.710
DLCO % b	−0.185	0.366	0.163	0.425	−0.362	0.070
DLCO % f/b	−0.207	0.309	−0.145	0.480	−0.090	0.662
Lung volume b	0.102	0.578	−0.582**	0.000**	−0.490**	0.004**
Lung volume f/b	−0.474**	0.006**	−0.200	0.272	−0.422*	0.016*
Mean lung density b	0.020	0.912	0.523**	0.002**	0.570**	0.001**
Mean lung density f/b	−0.469**	0.007**	−0.122	0.507	−0.402*	0.022*

## Discussion

In this study, we propose a two-phase dual-energy CT-based method allowing for assessment of regional lung function in addition to morphology in patients with IPF in a routine clinical setting.

In addition to the morphological assessment of the lung parenchyma, this method creates data for assessment of regional ventilation, lung perfusion, and late enhancement using standard dual-energy-source CT equipment. Our correlation analysis demonstrated the potential of the image-derived functional information as a marker for future progression of fibrosis. An increase of regional ventilation and late enhancement preceded future progression of fibrosis as measured by loss of lung volume and increase of lung density.

Increased mean regional ventilation correlated with the future decline in lung function, loss in lung volume, and increase in mean lung density, all of them indicating the progression of fibrosis. Interestingly, there was no correlation of mean regional ventilation with the baseline FVC%. One possible explanation is the limitation of the regional ventilation calculation to the functional lung volume, while the FVC% is a global parameter that includes the entire lung with potentially existing highly pathological areas (emphysema, advanced fibrosis, etc.). We observed that most patients had unphysiologically high regional ventilation areas with values exceeding 0.75 (see Fig. 3), while in the literature, values between 0.4 and 0.5 have been described for Jacobian-derived mean ventilation [19] in healthy volunteers. Transferred to PFT measurements, though measured globally, a regional ventilation value exceeding 0.75 corresponds to a pathologically low residual volume < 25% of total lung capacity in these areas.

Almost 50 years ago, it was described that micromechanical anomalies caused by alveolar instability could explain regionally increased ventilation within the functional lung tissue [23]. In their model of alveolar interdependence, Mead and co-workers assigned collapsed alveoli the role of stress concentrators. This mechanism has been proposed very recently as an important contributor to the development of fibrosis [24, 25]. Collapsed alveoli affecting neighboring inter-alveolar septa act as outward tethering forces so that open alveoli, which are adjacent to a cohort of collapsed alveoli, are exposed to potentially harmful overdistension and, therefore, regional hyperventilation [24, 25].

For lung perfusion, we saw a negative correlation with FVC%, lung volume, and mean lung density at baseline, but no correlation with their ratios. Lung perfusion, therefore, appears to be of limited use as a predictive imaging marker, but it seems to reflect the extent of parenchymal changes at baseline. Moon et al showed similar results describing a negative correlation of the mean iodine value of the total lung volume with FVC [26]. High values of perfusion might indicate increased disease activity. In that context, Ackermann et al described neoangiogenesis in fibrosing regions [27].

Similar to regional ventilation, we found a correlation of high late enhancement values with signs of progressive lung fibrosis. Considering that late enhancement is measured in lung tissue, that contains a substantial amount of air, values above vascular contrast (> 100% as measured in vessels in the delayed scan), as found in some regions of the lungs in most patients, inevitably imply a pathological condition. Analogous to the findings of late enhancement in a myocardial scar in MRI, we suspect that the increased enhancement indicates capillary leakage [28, 29] in these regions, which leads to contrast enhancement in the extravascular interstitium.

The fact that all three functional parameters of regional ventilation, perfusion, and late enhancement were significantly lower in areas of normal lung attenuation (NAA, - 950 to - 600 HU) compared to high-attenuation areas (HAA, - 600 to - 250 HU) could indicate that HAA represents areas at risk in the process of progressive fibrosis.

The study suffers from several limitations.

First of all, the study group is rather small, and our study can, therefore, only be considered a feasibility study. Evaluation of this technique in larger patient groups, with longer time intervals and close correlation with clinical symptoms and CT morphology, is needed.

The change of PFT scores, mean attenuation, and lung volume was normalized to a 1-year time frame. We accepted the inevitable inaccuracy caused by the assumption of linear change over time because we think that normalized measures increased the comparability of patients in this rather small study group and, at the same time, improve the statistical assessment of the significance of correlations.

Regional ventilation and perfusion are—among other factors—dependent on patient position and gravity effects, a fact all imaging techniques have in common. More evaluation is needed to assess the dependence on position and breathing depth as well as the reproducibility of measurements.

In that context, it is important to consider that the calculation of the Jacobian determinant depends on the quality of the registration, which is very demanding due to the large deformation of the lung between full inspiration and full expiration and as such also represents a source of potential inaccuracy.

For late enhancement imaging, we used a delay of 5 min. The optimal delay time for late enhancement in the lungs is unknown. In studies evaluating late enhancement in cardiac CT, a delay time of 5 to 10 min is proposed [30]. We adopted the results from Lardo et al, who found a maximum late enhancement after 5 min in an animal model.

We only present the correlation between mean scores of CT-derived morphological and functional parameters. A region-based analysis is, of course, desirable and would improve the spatial correlation between regional ventilation, late enhancement, and morphological changes over time that way potentially contributing to the understanding of the underlying pathomechanism of progressive fibrosis or other diffuse parenchymal lung diseases. However, this kind of spatial registration is technically very demanding as inspiration and expiration CT scans at two-time points have to be aligned to a single reference scan and is therefore subject to further studies.

## Conclusion

The proposed ventilation-perfusion-late enhancement CT (VPL-CT) allows for a combined assessment of regional lung function and morphology with high spatial resolution in a routine clinical setting.

Regional ventilation and late enhancement are promising early imaging markers for longitudinal analysis of IPF patients preceding disease progression measured with PFT and follow-up CT.

## Abbreviations

DLCO: Diffusing capacity of the lung for carbon monoxide
FVC: Forced vital capacity
HAA: High-attenuation areas
IPF: idiopathic pulmonary fibrosis
LAA: Low-attenuation areas
NAA: Normal-attenuation areas
PBV: Pulmonary blood volume
PFT: Pulmonary function test
VNC: Virtual non-contrast
VPL-CT: Ventilation-perfusion-late enhancement CT

## Declarations

### Guarantor

The scientific guarantor of this publication is Hoen-oh Shin.

### Statistics and biometry

No complex statistical methods were necessary for this paper.

### Methodology

• retrospective

• observational

• performed at one institution
